# The McKissock's technique in reduction mammaplasty: A comparative study of outcomes and complications in 211 consecutive patients

**DOI:** 10.3389/fsurg.2022.970381

**Published:** 2022-11-10

**Authors:** Francesco Messana, Martina Grigatti, Valentina Budini, Federico Ricci, Tito Brambullo, Franco Bassetto, Vincenzo Vindigni

**Affiliations:** ^1^ Resident in Plastic, Reconstructive and Aesthetic Surgery, Padua University Hospital, Padua, Italy; ^2^ Consultant in Plastic, Reconstructive and Aesthetic Surgery, Padua University Hospital, Padua, Italy; ^3^ Chief of the Clinic and Full Professor of Plastic, Reconstructive and Aesthetic Surgery, Padua University Hospital, Padua, Italy; ^4^ Associate Professor of Plastic, Reconstructive and Aesthetic Surgery, Padua University Hospital, Padua, Italy

**Keywords:** breast reduction, reduction mammaplasty, post bariatric surgery, breast reshaping, mcKissock

## Abstract

Reduction mammaplasty is one of the most popular plastic surgery procedures requested by patients. The areola holding flap can be sculpted using a variety of methods that have evolved over time dependent on vascularity. Our institution has always employed the vertical bipedicle technique proposed by Mckissock, and we still favor it over other methods for larger breasts. In this study, we examined the case-study data from the Padua University Hospital's Unit of Plastic and Reconstructive Surgery from January 2009 to December 2021. The rate of complications among patients who received breast reduction using the McKissock technique and all other procedures carried out at our facility was compared. We identified 90 postoperative problems in all (affecting 42.65% of the patients) and categorized them using the Clavien Dindo system. The groups were comparable in age, BMI, and follow-up time. Similar findings emerged from the study of the single groups' complication rate. The statistical analysis did not reveal any appreciable variation in total complications or scar quality across groups. Therefore, in order to guarantee NAC survival, a stable shape, and a full upper pole, we think it is preferable to bind more than one pedicle in cases of very large breasts. Based on the results of our experience, we also recommend the McKissock approach as the first option for patients with large and ptotic breasts, particularly those who have undergone bariatric surgery and need a full upper pole and a stable outcome.

## Introduction

Reduction mammaplasty is one of the most common procedures in plastic surgery. Because of its aesthetic and functional purposes, this surgery has relevant implications for the patients who, most of the time, witness a consistent improvement in their quality of life (1).

According to the Literature, it is estimated that over the last tens of years many authors have described over 50 variations of the original technique (2). The variances are due to the choice of pedicle for the Nipple-Areola Complex and various skin design patterns (NAC). The fundamental goal of all of these diverse strategies is to reduce the breast in an appropriate and proportionate manner while maintaining high viability and sensitivity of the NAC.

The different techniques are usually named according to the location of the base of the pedicle, as it can be superior, inferior, medial, lateral, central, or a combination of them (3).

In 1972, Paul McKissock described the vertical bipedicle technique. The main benefit introduced by this technique was the inclusion of perforators from the superior and inferior poles of the breast. Before that time, the removal of the upper pole of the breast frequently resulted in vascular issues with the NAC, such as inadequate blood flow or venous congestion (4, 5).

Many older procedures have been abandoned in recent years because of the high number of complications such as skin/nipple necrosis and loss of sensibility of the NAC.

In the majority of centers throughout the world, McKissock's approach has mostly been replaced by her direct evolution, the inferior pedicle procedure described by Robbins (6).

At our Institution, we have always performed the vertical bipedicle technique for reduction mammaplasty, and it still represents our first choice for larger breasts.

In this study, we conducted a retrospective analysis of the case history of the Padua University Hospital's Unit of Plastic and Reconstructive Surgery. To further evaluate the present role of this approach, we wanted to compare the complication rate between patients who underwent breast reduction using McKissock's technique and those on whom we selected different pedicles.

## Materials and methods

### Patients and preoperative assessment

A retrospective analysis was carried out on the breast reductive surgery performed at Padua University Hospital (Italy) from January 2009 to December 2021. We conducted our investigation using the clinic's database, patient records, outpatient clinic reports, and photo archives.

All of our adult female patients with bilateral involvement and a history of hypertrophic breast of any cause were included. Breast cancer history, unilateral involvement, and follow-up dropout were considered exclusion criteria.

Since we operate in a public health care facility, before surgery all of our patients had to undergo a mammogram or breast ultrasound, a nutritional and endocrinological assessment, as well as a physiatric or orthopedic assessment to certify any grade of musculoskeletal or skin disorder caused by the hypertrophic breasts (e.g, chronic dorsalgia, spine deformity, intertrigo, inframammary ulcerations, etc.). In addition, for patients with a history of obesity, we requested a stable weight for at least 6 months for eligibility.

During the preoperative evaluation, we recorded the patient's complete medical history and BMI and collected preoperative photographs. We also acquired informed consent.

### Surgery and in-hospital management

On the day of surgery, all the patients received both antibiotic and antithrombotic prophylaxis. We routinely use first-generation cephalosporins (clindamycin or macrolides when there is a history of penicillin allergy) 30 min before surgery and continue for the following 48–72 h. As regards thromboprophylaxis, patients routinely wear anti-embolism stockings before the surgical procedure and up to 10 days post-op. In the case of increased thrombotic risk, we administer 4000 UI of enoxaparin daily from the day after surgery to 7–10 days post-op. The skin markings were taken in a standing position before surgery and we used an inverted T type of design on every patient. During surgery, the removed breast parenchyma was weighed and the data was recorded in the operative report. The Pathologist routinely analyzed the removed tissue for the detection of any undiagnosed tumoral focus. Drains were kept in place until they collected less than 50 cc/day.

### Follow-up and outcome evaluation

**Figure 1:**
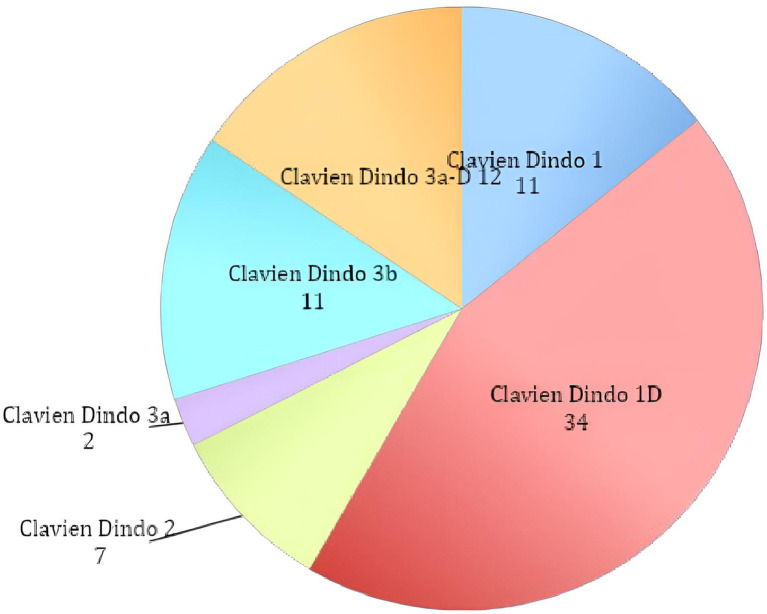
Total complications classified according to the clavien-dindo classification.

Follow-up visits were scheduled at 7–14–30 days and 3–6–12 months after discharge. All patients included in the study followed the same follow-up. Some of the more complex cases in the study needed additional visits after the last scheduled follow-up, and we still included them in the study. Patients who did not attend scheduled visits were excluded from the study. We analyzed the postoperative outcomes and complications recorded during follow-up visits and collected the latter according to the Clavien-Dindo classification (7) (Figure 1). If more than one complication had occurred in a given patient, we considered only the more severe one (higher in the Clavien-Dindo classification). As proposed by Clavien et al., we added the suffix “D” whenever the complication occurred after the patient's discharge or left a permanent disability. We also evaluated the quality of the scars using a scale that ranges from 0 to 3. We rated 0 scars that needed a surgical revision, 1 if they were both hyperchromic and hypertrophic, 2 if they presented hypertrophy or hyperchromasia, and 3 in the case of an optimal, normochromic, and normal-trophic scar.

### Statistical analysis

In order to do the statistical analysis, we separated our patients into two groups: “Group A” comprised all the patients treated with the McKissock technique, and “Group B”, was made up of the patients who underwent breast reduction using other techniques.

IBM® SPSS® 12 Statistics 24 (IBM, Armonk, NY, United States) was used for statistical analyses. We used a two-tailed Student *t*-test for means comparison of continuous values and a *χ*2 test for categorical data. We considered 0.05 for statistical significance.

### Ethical approval

Our ethics committee did not require formal ethical approval for retrospective reviews.

## Results

From our research, of the 251 patients who had undergone breast reduction, 40 did not meet the inclusion criteria and were excluded from the study.

The total mean age was 48 years (range 18–77; SD 13) and the total mean BMI was 28.97 (range 20.1–44.6; SD 4.76). 30 patients were smokers, while 9 patients had type 2 diabetes mellitus. The mean weight of the breast parenchyma removed (right plus left mammary parenchyma) was 1376,77 (SD 999.23).

We detected a total of 90 (42.65% of the patients) postoperative complications and classified them according to the Clavien Dindo classification (Graph 1; Table 1 for details).

**Table 1:** Detailed total complications.

	McKissock	Others	Total
**Clavien Dindo 1**	**4**	7	**11**
Hematoma	3	3	6
NAC venous congestion	0	2	2
Upper limb nerve stupor	0	1	1
Amoxicillin related urticaria	1	0	1
Seroma	0	1	1
**Clavien Dindo 1-D**	**23**	11	**34**
Wound dehiscence	7	4	11
Fat necrosis	6	2	8
Sutures reject	0	1	1
Delayed wound healing	10	4	14
**Clavien Dindo 2**	**4**	**3**	**7**
Post-operative anemia	4	2	6
Mondor's syndrome	0	1	1
**Clavien Dindo 3a**	**1**	**1**	**2**
Wound dehiscence	1	1	2
**Clavien Dindo 3a-D**	**8**	**4**	**12**
Pathological scar	5	2	7
Dog ears	2	1	3
Fat necrosis	0	1	1
NAC partial necrosis	1	0	1
**Clavien Dindo 3b**	**7**	**4**	**11**
Hematoma	4	4	8
Cutaneous fistula	2	0	2
Surgical site infection	1	0	1
**Clavien Dindo 3b-D**	**6**	**7**	**13**
Pathological scar	2	1	3
Dog ears	0	2	2
Insufficient reduction	1	0	1
Wound dehiscence	1	1	2
NAC partial necrosis	1	0	1
Fat necrosis	0	1	1
Late abscess	0	1	1
Breast asymmetry	1	0	1
Recurrent ptosis	0	1	1

As mentioned above, Group A included all the patients treated with the McKissock technique, while Group B included patients who underwent breast reduction using alternative approaches. Of them, 59 had a superior pedicle, 18 were inferior, 5 were superomedial, 1 was medial, and 5 underwent amputation and NAC autograft according to Thorek.

The groups were comparable for age, BMI, and follow-up time, but the total breast removed was higher in Group B (Table 2).

**Table 2:** Group means comparison for age, BMI, follow-up time and total breast removed. 0.05 was considered for statistical significance.

Means	Group A	Group B	*p* value
Age (years)	49	49	0.096
BMI (kg/m^2^)	29,04	28,80	0.059
Follow-up time (months)	6	5	0.619
Breast removed (g)	1171,93	1661,88	0.001

The analysis of the complication rate of the single groups showed similar outcomes, as we registered 53 cases in Group A (43.09%) and 37 in Group B (42.05%) (Graph 2; Graph 3).

The statistical analysis did not show any significant difference between groups regarding total complications; no statistical differences were registered in any of the Clavien Dindo sub-groups (Table 3).

**Table 3:** Statistical comparison between complication sub-groups. 0.05 was considered for statistical significance.

Clavien Dindo	Group A	Group B	*p*-value
Clavien Dindo 1	4	7	0.196
Clavien Dindo 1-D	23	11	0.227
Clavien Dindo 2	4	3	0.949
Clavien Dindo 3a	1	1	0.811
Clavien Dindo 3a-D	8	4	0.545
Clavien Dindo 3b	7	4	0.712
Clavien Dindo 3b-D	6	7	0.359
Total	53	37	0.879

Finally, the mean quality score of the scars was 2.417 for Group A and 2.442 for Group B, showing no statistically significant difference between groups (*p* = 0.859).

## Discussion

Reduction mammaplasty is one of the most performed procedures in plastic surgery worldwide (1). It is mostly indicated to treat post-pregnancy hypertrophy, virginal hypertrophic breast, and patients who have undergone significant weight loss with residual hypertrophic breasts. In the past decades, many authors have described different techniques for breast reduction but none of them has been consecrated as the “Gold Standard” by the scientific community.

The patient's medical history, beginning breast size and shape, desired final size, and surgeon preference all play a significant role in this.

Despite the aforementioned initial variety, the principal purposes of the procedure are to achieve a balanced and consistent reduction over time, a natural-looking shape with good projection, and to safeguard the viability of the NAC.

Nevertheless, despite the multiple approaches documented in the literature and technological developments, the incidence of complications in the reduction of mammaplasty still remains between 5% and 50% overall (8).

In our retrospective analysis, we found a total of 90 (42,65% of the patients) postoperative complications according to the Clavien-Dindo classification. The statistical analysis did not show any significant difference between groups regarding total complications (43,09% vs. 42,05%, *p* = 0.87). For both groups, the most represented complication was type 1-D (23 vs. 11 cases). In the patients treated with the McKissock technique, the second most represented complication was the IIIa-D (8 cases), while in the patients operated with other techniques were the types 1 and 3b-D (7 cases both). We did not record any complications of types 4 or 5 in either group.

Despite the relatively high total rate of complications, 57.77% were type 1, 1-D, and 2, meaning that these patients did not receive any surgical treatment. Of the remainder, 15.6% of the complications were type 3a/3a-d, and the patients underwent only minor revisions under local anesthesia. Finally, 26.6% of the complications were type 3b/3b-d and required revision surgeries under general anesthesia.

**Figure 2:**
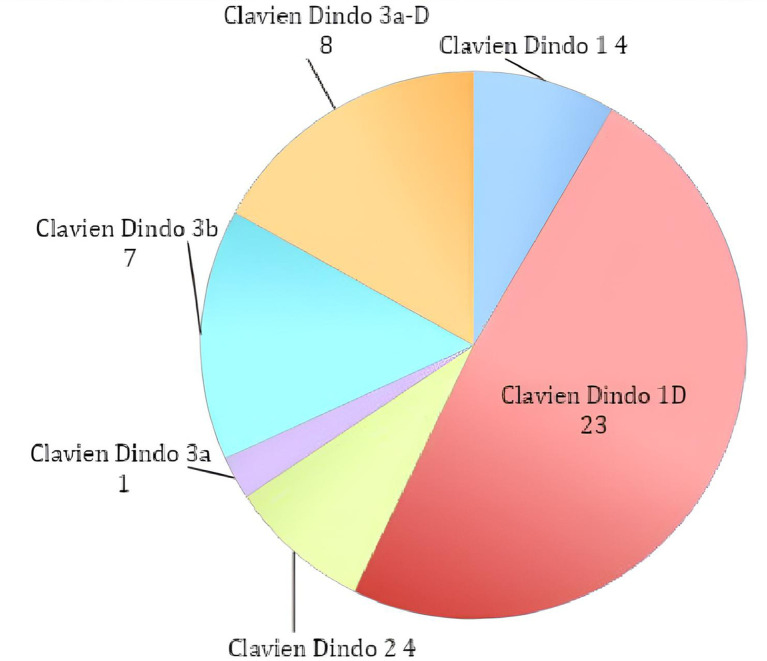
Complication sub-classification of group A.

**Figure 3:**
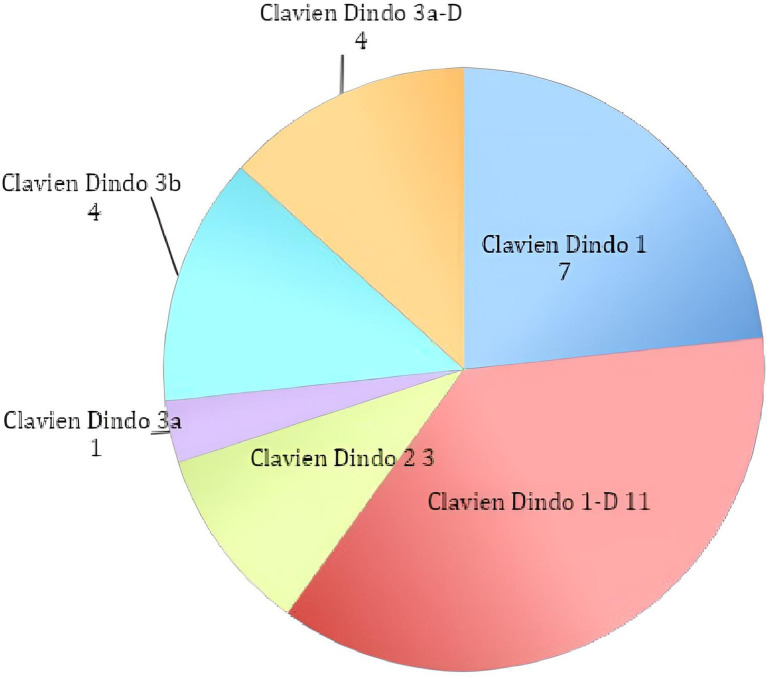
Complication sub-classification of group B.

Additionally, we discovered no evidence of a statistically significant difference between the subtypes of complications in either group, and our findings are consistent with the rates published in the literature, supporting the non-inferiority of the McKissock approach over the alternatives (Figures 2 and 3).

Nevertheless, we must state that one of the limitations of our study is the difference in the volume of breast tissue removed between the two groups. In particular, the group subjected to breast reduction surgery with the McKissock technique was found to have removed about 400 g less than the other group. This may be because patients who underwent operations using the Thorek technique were included in group B, but it is still a limit that must be carefully taken into account when evaluating the outcome.

After McKissock first described the vertical bi-pedicled flap in 1972, different authors proposed modifications to the original techniques like augmentation auto-flaps and design variations (9, 10).

However, as single pedicle procedures (such as superior or inferior pedicles) gained popularity, the bi-pedicled flap described by McKissock gradually lost favor with most surgeons worldwide. In this regard, we performed a bibliographic search on the Pubmed platform, considering the indexed scientific articles. Since 1982, only 10 papers have been published that contain the word “Mc Kissock” in the text, and their number has drastically reduced compared to the previous forty years. On the contrary, the total number of publications concerning all breast reduction operations has progressively increased (3,354 articles since 1982).

As we consider the anatomy of the breast, the vascular supply comes from the internal mammary vascular system with a contribution from the superficial branch of the lateral thoracic artery and some branches of the thoracoacromial pedicle.

**Figure 4:**
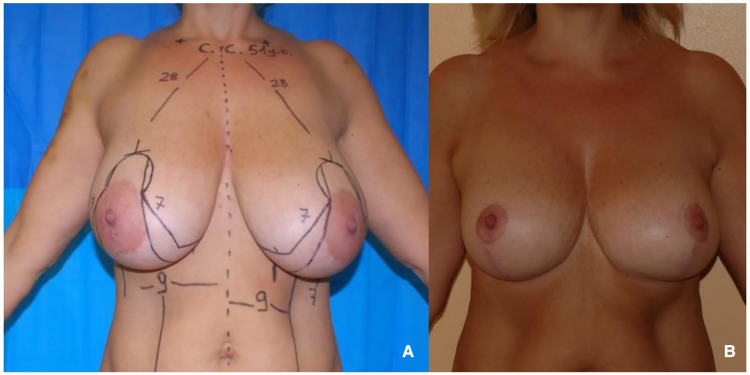
(**A**) 51 years old patient with virginal hypertrophic breasts. (**B**) 1-year follow-up after McKissock's reduction mammaplasty.

The McKissock's reduction mammaplasty preserves two of the main pedicles that bring blood to the NAC. Those are: 1) the descending artery, which comes from the internal mammary system and is included in the superior pedicle; 2) the deep arterial system, coming from the fourth intercostal space and is included in the inferior pedicle (11) (Figure 4).

The McKissock resection plan also has the benefit of preserving the venous system, which often drains through the superior dermal-glandular flap.

Preserving the viability of the NAC and minimizing the occurrence of venous congestion depends in large part on the possibility of having a double pedicle arterial flow and maintaining the venous drainage.

**Figure 5:**
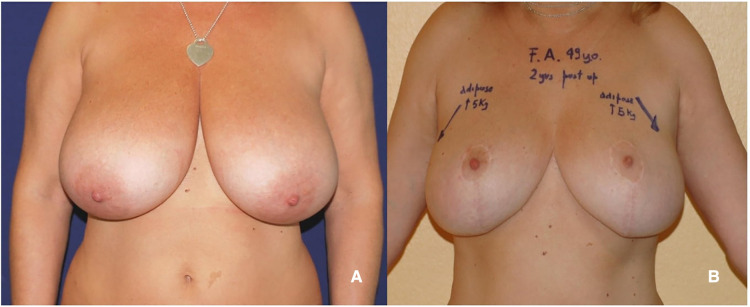
(**A**) 49 years old patient with hypertrophic breasts after weight loss. (**B**) 2 years follow-up after McKissock's reduction mammaplasty.

Moreover, 24.17% of the total patients and 26.83% of Group A had a history of massive weight loss. As reported by Polotto et al., one of the main features of a post-bariatric breast is an empty upper pole (12) (Figure 5).

By folding the upper portion of the pedicle into the upper quadrants, the McKissock approach offers these patients an excellent filling and reshaping of the upper pole.

Additionally, Clavien-Dindo classification was selected since it is a systematic method for assessing surgical complications and prevents any potential misunderstandings. In truth, terminology like “mild” and “major” complications are common in publications, but they are frequently arbitrary and do not allow for a trustworthy comparison of complication rates. In addition, we can further specify that the Clavien-Dindo complications 1 and 2 can be defined as non-operative, whereas those from 3 to 5 lead to some kind of surgical or intensive medical treatment.

**Figure 6:**
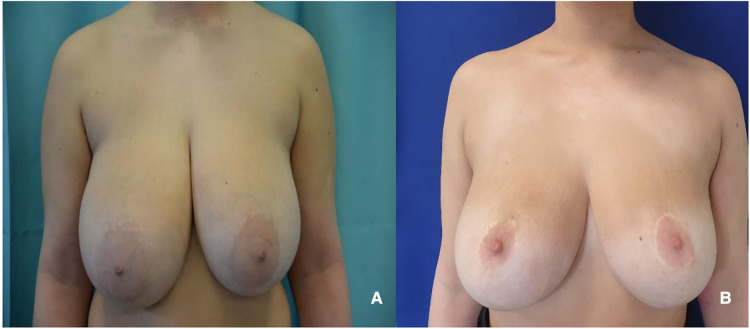
(**A**) A 24-year-old patient with virginal hypertrophic breasts. (**B**) 21 months follow-up after McKissock's reduction mammaplasty.

In conclusion, based on our observations, it is preferable to secure multiple pedicles while working on very large breasts in order to guarantee the NAC's viability, a stable shape, and a full upper pole. The chance of the lower pole bottoming out is also reduced by the potential for securing the folded upper section of the dermal-glandular flap to the second or third rib. In light of the outcomes of our case study, we, therefore, recommend the McKissock approach as the first option for patients with large and ptotic breasts, particularly those who have recently undergone bariatric surgery and need a full upper pole and a stable outcome (Figure 6).

## Data Availability

The raw data supporting the conclusions of this article will be made available by the authors, without undue reservation.
